# Paths towards parenthood after repeated treatment failures: a comparative study on predictors of psychological health outcomes in infertile couples persisting in treatments or opting for adoption

**DOI:** 10.3389/fpsyg.2023.1147926

**Published:** 2023-06-05

**Authors:** Maria Clelia Zurlo, Maria Francesca Cattaneo Della Volta, Federica Vallone

**Affiliations:** ^1^Dynamic Psychology Laboratory, Department of Political Sciences, University of Naples Federico II, Naples, Italy; ^2^Department of Humanities, University of Naples Federico II, Naples, Italy

**Keywords:** infertility, adoption, coping strategies, couple’ s dyadic adjustment, repeated treatment failures, psychological health

## Abstract

**Introduction:**

Infertility literature suggests widespread recourse to long-term medical treatments despite evidence of high stress, costs, and adverse effects of repeated treatment failures. However, there is a lack of research comparing predictors of stress and psychological health outcomes between members of infertile couples who – after repeated failures – persist in pursuing medical treatments (PT) with those who opted for quitting treatments and adopting (QTA). Basing on a transactional and multidimensional approach to infertility-related stress and health, the present study aims at exploring individual (socio-demographics; coping strategies) and situational (infertility-related parameters; infertility-related stressors; couple’s dyadic adjustment dimensions) predictors of state-anxiety and depression in male and female partners of PT-infertile couples and of QTA-infertile couples.

**Methods:**

Participants were both members of 176 couples with duration of infertility and a history of medical treatments for at least 3 years (76 PT-infertile couples, 100 QTA-infertile couples). The study variables were compared by study group across genders. Structural equation models (SEM) were used to test main and moderating effects of study variables on state-anxiety and depression by study group and across genders.

**Results:**

Members of infertile couples quitting treatments and adopting (QTA) reported significantly lower levels of state-anxiety and depression, higher stress related to need for parenthood and rejection of childfree-lifestyle and lower stress related to social and couple’s relationship concerns than those who persist in pursuing medical treatments (PT). Members of infertile couples quitting treatments and adopting (QTA) recurred to a greater extent to active coping strategies (problem-solving/social-support) and to a lower extent to passive coping strategies (avoiding/turning-to-religion), and they reported higher levels of dyadic adjustment. Specificities in main and moderating factors related to state-anxiety and depression by study group and across genders were found.

**Conclusion:**

Findings should be addressed to provide a comprehensive assessment of both members of infertile couples facing repeated treatment failures to identify risks and resources and develop tailored evidence-based interventions.

## 1. Introduction

Infertility is defined as the inability to achieve a clinical pregnancy after 12 months or more of regular, unprotected, sexual intercourse (Zegers-Hochschild et al., 2017). It has been estimated to affect about 15% of reproductive-aged couples worldwide (World Health Organization [WHO], 2023), and it represents a significant life crisis (Ying et al., 2015; Tiu et al., 2018; Hocaoglu, 2019) that may have a profound impact on psychological health (Fassino et al., 2002; Peterson et al., 2014; Masoumi et al., 2019; Abdishahshahani et al., 2020), as well as on sexual, marital and social life (Hasanpoor-Azghady et al., 2019; Sahin and Gursoy, 2021; Boivin et al., 2022).

However, given the advancements in the medical field, an increasing number of infertile couples can fulfill their desire to have a child through the recourse to assisted reproductive technologies (ART). Indeed, research has indicated a considerable percentage of *in-vitro* fertilization (IVF) treatment success (Human Fertilization and Embryology Authority [HFEA], 2019). In particular, the average live-birth rate after a single treatment is up to 32% (patients <35 years old) and despite it steadily decreasing with each new cycle – also due to the growing patient age – the birth rate remains at about 20% for the first three cycles (Human Fertilization and Embryology Authority [HFEA], 2018, 2019).

Nevertheless, although IVF provides many infertile couples with a chance to accomplish parenting wishes, this frequently results in stressful and invasive treatments and in a long path marked by repeated failures (Daniluk, 2001; Maroufizadeh et al., 2015; Ho et al., 2020). Approximately 25% of patients have experienced repeated implantation failures (Coughlan et al., 2014), and couples often undertake more than five IVF cycles (Simonstein et al., 2014). However, research has also demonstrated that as the number of unsuccessful cycles and duration of infertility increase, the success rate falls, so that 30% of patients undergoing medical treatments do not achieve parenthood (Gameiro and Finnigan, 2017).

Infertility literature widely highlighted that diagnosis and treatments are very stressful experiences and can have a deep impact on psychological health conditions and quality of life. This is particularly true when couples experience a long duration of infertility and repeated infertility treatment failures (Karaca et al., 2016; Gameiro and Finnigan, 2017; Zurlo et al., 2018; Ni et al., 2022). From this perspective, research targeting infertile couples persisting in medical treatments suggested that long-lasting infertility experiences and repeated unsuccessful treatment cycles may result in chronic stress and increasing perception of loss of behavioral/emotional control, as well as sexual dysfunctions and worse pregnancy rates (Ragni et al., 2005; Verhaak et al., 2005; Dong et al., 2021). Furthermore, the higher the length of infertility experience, the higher the risk for infertile couples to report significant levels of stress and psychological disease, mainly in terms of anxiety and depression (Maroufizadeh et al., 2015; Gameiro and Finnigan, 2017; LoGiudice and Massaro, 2018), and worse quality of life (Boivin et al., 2011; Ozkan et al., 2015). In line with this, research emphasizing the detrimental impact of lengthened duration of infertility and repeated treatment failures has also recognized 3 years from diagnosis and experienced failures as a crucial moment to be considered, not only in terms of decreasing the chance of pregnancy rates (Akande et al., 2004) but also in terms of worsening of psychophysical health conditions (Domar et al., 1992; Seok Kee et al., 2000; Turan et al., 2014) and impairment of infertile patients’ stress-and-coping processes, resulting in exacerbation of perceived stress and severe damages of individuals’ adjustment resources (Zurlo et al., 2018).

When confronted with the reality of repeated treatment failures, the couples are therefore forced to revise and re-assess their needs and desires for a child and parenthood at individual and couples levels (Daniluk, 2001; Throsby, 2004). Indeed, it should be noticed that all infertile couples reported their need to try everything they could to have a biological child (Lockerbie, 2014; Park and Wonch Hill, 2014) but they also need to face – at some stage – a critical point, in which the burden, the stress, and the distress linked to repeated failures impose a reflection and require the couple to make a choice about medical treatments (Daniluk and Hurtig-Mitchell, 2003). The decision-making process induces some infertile couples to opt for quitting medical treatments and remaining childless or achieving parenthood by adoption, while other couples persist in pursuing treatments and display high reluctance to stop them despite the increasing burden and negative effects at individual and couple levels (Lockerbie, 2014; Park and Wonch Hill, 2014).

Research comparing psychological health conditions reported by infertile couples with fertile and adoptive couples revealed the infertile group reported significantly higher levels of shame, anxiety, and depression (Galhardo et al., 2011), and clinicians frequently compared the persistence in pursuit of assisted reproductive technologies (ART) to addictive behavior (Visigalli, 2011; Abramov et al., 2022). Moreover, research targeting motivations driving couples’ persistence in medical treatments and their negative attitudes toward adoption underlined the role of perceived risk to have a difficult child due to a difficult past (van Balen et al., 1997), concerns linked to blood tie preservation and fear of genetic diseases (Miall, 1987; Goldberg et al., 2009; Petropanagos, 2010), need of keeping infertility a secret (Bharadwaj, 2003), racial prejudice (Rolnick and Pearson, 2017), concerns linked to the age of the adopted child (Grattagliano et al., 2012), and anticipatory regret (Sandelowski et al., 1991).

Furthermore, research targeting adopting couples often reported they needed some time to recover from prolonged treatments and to reassess/reconsider the role of parenting in their lives before starting the adopting path, which is considered as a backup plan, rather than a choice parallel to undertaking treatments (Daniluk and Hurtig-Mitchell, 2003).

Nevertheless, all these studies targeted single dimensions and/or were descriptive/qualitative in nature. They neither analyze the impact of specific predictors nor identify potential risk and protective factors (main and moderating effects) influencing infertility-related stress process and psychological health outcomes as well as the connected decision of persisting in pursuing medical treatments (PT) or quitting treatments and opting for adoption (QTA) in a comprehensive transactional/multidimensional perspective.

The transactional theory of stress defines the stress process as depending on the interplay between situational dimensions (i.e., perceived stressors) and individual dimensions (i.e., individual and personality characteristics and adopted coping strategies) (Lazarus and Folkman, 1984). In this direction, in the last decades, research has increasingly addressed the psychosocial challenges of infertility diagnosis and treatments, identifying, on the one side, infertility-related stressors (i.e., social concerns, relational and couples concerns, need for parenthood, and rejection of childfree lifestyle), which were all well-demonstrated to play a key role in impacting psychological health in members of infertile couples (Newton et al., 1999; Lakatos et al., 2017; Galhardo et al., 2020). On the other side, research has identified specific potential individual protective factors (i.e., adopted coping strategies; Benyamini et al., 2008; Zurlo et al., 2020a,b; Cattaneo Della Volta et al., 2022), and relational protective factors (i.e., perceived couple’s dyadic adjustment; Cserepes et al., 2013; Zurlo et al., 2019; Iordachescu et al., 2021), which may promote infertile couples’ well-being.

Basing on a transactional and multidimensional approach, all the above-mentioned key variables reported, independently, in the infertility literature have also been simultaneously considered in a statistically valid predictive infertility-related stress model along with socio-demographic characteristics (i.e., age, educational level, employment status) and infertility-related parameters (i.e., type of diagnosis, duration of infertility and repeated treatments) (Zurlo et al., 2020a). This model allows accounting for the complex effects – not only main but also interplay effects – of a wide set of individual predictors (i.e., socio-demographic characteristics and coping strategies) and situational predictors (i.e., infertility-related parameters, perceived infertility-related stressors, and perceived dyadic adjustment dimensions) of psychological health outcomes in both members of infertile couples.

The adoption of this approach provides the researchers and the clinicians with the possibility to identify not only main risk and protective factors, but also those moderating factors (Lorah and Wong, 2018; Liw and Han, 2020), namely factors able to effectively counteract, buffer and prevent – or conversely to exacerbate significantly – the detrimental effects of perceived stress, i.e., individual characteristics (i.e., socio-demographic and coping strategies; Jordan and Revenson, 1999; Berghuis and Stanton, 2002; Peterson et al., 2006, 2008; Kraaij et al., 2009; Zurlo et al., 2018, 2019), infertility-related parameters and relational resources (i.e., perceived couple’s dyadic adjustment) (Peterson et al., 2003; Monga et al., 2004; Onat and Beji, 2012; Cserepes et al., 2013; Ying et al., 2015; Zurlo et al., 2018, 2019) which are all well-recognized as serving this pivotal role.

Therefore, since offering tailored support to patients to adjust to unmet parenthood wishes is widely considered a primary goal for optimal IVF management (Gameiro et al., 2013), the present study is based on the evidence provided within infertility research and on the predictive infertility-related stress model described above (Zurlo et al., 2020a) and aims to reflect upon the stress and health processes for infertile couples who, after at least 3 years of duration of infertility and repeated treatment failures, were attempting to achieve parenting goals, or by opting to persist in treatments or, instead, by opting for quit treatments and embarking on a different path to achieve parenthood, namely the adoption.

Specifically, the present study aims to preliminarily explore whether there was any difference between male and female partners of infertile couples who persist in pursuing medical treatments (PT) in comparison to members of infertile couples who opted for quitting treatments and adopting (QTA).

Furthermore, it aims at testing the main and interacting (moderating) effects of all the above-mentioned individual and situational factors potentially influencing infertility-related stress and health process in both members of PT and QTA infertile couples. This would allow reaching a greater understanding of these two paths to achieving parenthood after prolonged infertility and repeated treatment failures. It was indeed sought to understand and compare the experiences of patients who persist in treatments – despite its detrimental impact – and of those who, differently, can access the choice of quitting treatments and opt for alternative paths for achieving parenthood.

In line with the study aims and given the scarcity of empirical studies of comparative nature in this field, the following research questions, rather than formal hypotheses, have been proposed and originally tested:

*Research question one (RQ1)*: Are there differences in perceived levels of state-anxiety and depression reported by male and female members of infertile couples who – after at least 3 years of duration of infertility and repeated treatment failures – persist in pursuing medical treatments (PT) in comparison to members of infertile couples who opted for quitting treatments and adopting (QTA)?

*Research question two (RQ2)*: Are there differences in socio-demographics and infertility-related parameters (*RQ2.a*), in perceived levels of Infertility-related stress dimensions (*RQ2.b*), in recourse to coping strategies (*RQ2.c*), and in perceived levels of couple’s dyadic adjustment (*RQ2.d*) reported by male and female members of infertile couples who – after at least 3 years of duration of infertility and repeated treatment failures – persist in pursuing medical treatments (PT) in comparison to members of infertile couples who opted for quitting treatments and adopting (QTA)?

*Research question three (RQ3)*: Are there differences in the associations between socio-demographics and infertility-related parameters (*RQ3.a*), infertility-related stress dimensions (*RQ3.b*), adopted coping strategies (*RQ3.c*), and perceived levels of couple’s dyadic adjustment (*RQ3.d*) with perceived levels of state-anxiety and depression reported by male and female members of infertile couples who – after at least 3 years of duration of infertility and repeated treatment failures – persist in pursuing medical treatments (PT) in comparison to members of infertile couples who opted for quitting treatments and adopting (QTA)?

*Research question four* (*RQ4*): Do socio-demographics and infertility-related parameters (*RQ4.a*), adopted coping strategies (*RQ4.b*), and perceived levels of couple’s dyadic adjustment (*RQ4.c*) serve as significant moderators of the relationship between infertility-related stress dimensions and perceived levels of state-anxiety and depression across the two study groups and across gender (PT and QTA)?

## 2. Materials and methods

### 2.1. Participants and sampling

The present cross-sectional study aimed at reaching a greater understanding of two specific paths for achieving parenthood after at least 3 years of duration of infertility and ART treatments, namely persisting in treatments (PT) or opting for quitting treatments and adopting (QTA). Therefore, the sampling was limited to infertile couples with both duration of infertility and a history of ART treatments for at least 3 years. The study was conducted in Italy, in 2019, before the COVID-19 pandemic. Infertile couples still persisting in medical treatments (PT) were recruited from centers of assisted reproduction, and were all still undergoing ART treatments cycles. Couples who opted for quitting treatments and adopting (QTA) were recruited from foster care and adoption agencies. QTA couples were all undergoing the adoption process (i.e., they were at different stages but no couple had completed it and achieved foster parenthood), and all had quitted medical treatments (i.e., none of them was still undertaking ART treatments).

Chairmen were asked to give the authorization for administering a questionnaire in their centers/agency and, after obtaining their adhesion to the project, infertile couples were directly asked to participate in the study by one of the authors (researcher and psychologist). As inclusion criteria, couples should possess the following characteristics: (a) primary infertility; (b) duration of infertility for at least 3 years; (c) history of ART treatments for at least 3 years. The agreement by both members of the couple to participate in the study was also among the inclusion criteria. Indeed, if one partner refused to participate/failed to complete the survey, the couple was not included in the final dataset. Overall, 120 members of PT-infertile couples (120 men, 120 women) and 120 members of QTA-infertile couples (120 men, 120 women) were asked to individually complete a questionnaire lasting 15–20 min (one session), and one of the authors was present to answer any queries raised by participants. All the subjects were fully informed about the purpose of the study. They were assured about the confidentiality of the data, and they were informed that the data would be used only for the aim of the research. The project was approved by the Ethical Committee of Psychological Research of the University of Naples Federico II (IRB:34/2019). Research was conducted in accordance with the 1964 Helsinki Declaration and its later amendments or comparable ethical standards. Every precaution was taken to protect the privacy of participants and the confidentiality of their personal information, and the questionnaires were completed anonymously. Informed consent was obtained from each subject prior to participating in the study. Overall, 176 infertile couples with at least 3 years of both duration of infertility and a history of repeated ART treatment failures agreed to participate in the study, of whom 76 were members of PT-infertile couples (response rate: 63%) and 100 were members of QTA-infertile couples (response rate: 83%).

### 2.2. Measures

The questionnaire included a section dealing with background information, containing questions on socio-demographic characteristics and infertility-related parameters, along with valid tools for measuring infertility-related stressors, coping strategies, couples’ dyadic adjustment dimensions, and psychological health outcomes in terms of state-anxiety and depression.

#### 2.2.1. Socio-demographic characteristics

Socio-demographic characteristics were assessed by questions on gender (coded 0 = women; 1 = men), age (in years), educational level (coded 0 = upper secondary school; 1 = college) and employment status (coded 0 = unemployed; 1 = employed).

#### 2.2.2. Infertility-related parameters

Infertility-related parameters were assessed by using clinical records provided by the gynecologists, i.e., duration of infertility (in years), failed treatments (number), and type of diagnosis, namely female factor (coded 0 = no; 1 = yes), male factor (coded 0 = no; 1 = yes), combined factor (coded 0 = no; 1 = yes), and unexplained factor (coded 0 = no; 1 = yes).

#### 2.2.3. Infertility-related stress dimensions

Infertility-related stress dimensions were measured by using the Fertility Problem Inventory-Short Form (FPI-SF; Zurlo et al., 2017), which consists of 27 items on a 6-point Likert scale ranging from one (strongly disagree) to six (strongly agree) divided into four subscales, namely social concern, need for parenthood, couple’s relationship concern, rejection of childfree lifestyle. Social concern measures perceived stress related to comments and reminders of infertility and to feelings of social isolation (10 items; Cronbach’s α = 0.88; e.g., “Family get-togethers are especially difficult for me”); need for parenthood measures perceived stress related to viewing parenting as an essential life goal (6 items; Cronbach’s α = 0.88; e.g., “For me, being a parent is a more important goal then having a satisfying career”); couple’s relationships concern measures perceived stress related to decreased sexual enjoyment and to concerns about impact of infertility on quality of relationship (5 items; Cronbach’s α = 0.70; e.g., “My partner does not understand the way the fertility problem affects me”); rejection of childfree lifestyle measures perceived stress related to a negative view of living child-free/future happiness dependent on having a child (6 items; Cronbach’s α = 0.77; e.g., “having a child/another child is not necessary for my happiness”). In the present study, Cronbach’s α values were satisfactory; i.e., social concern (Cronbach’s α = 0.83); need for parenthood (Cronbach’s α = 0.82); couple’s relationship concern (Cronbach’s α = 0.80); rejection of childfree lifestyle (Cronbach’s α = 0.78).

#### 2.2.4. Coping strategies

Coping strategies were measured by using the Coping Orientation to Problem Experienced-New Italian Version (COPE-NIV; Carver et al., 1989; Sica et al., 2008), which consists of 60 items on a 5-point Likert scale ranging from one (I usually do not do this at all) to four (I usually do this a lot) divided into five subscales: social support (12 items; Cronbach’s α = 0.88; e.g., “I talk to someone to find out more about the situation”); avoiding (16 items; Cronbach’s α = 0.70; e.g. “I turn to work or other substitute activities to take my mind off things”); positive attitude (12 items; Cronbach’s α = 0.76; e.g. “I try to see it in a different light, to make it seem more positive”); problem solving (12 items; Cronbach’s α = 0.83; e.g. “I focus on dealing with this problem, and if necessary, let other things slide a little”); turning to religion (8 items; Cronbach’s α = 0.85; e.g. “I put my trust in God”). In the present study, Cronbach’s α values were satisfactory, i.e., social support (Cronbach’s α = 0.85); avoiding (Cronbach’s α = 0.84); positive attitude (Cronbach’s α = 0.79); problem solving (Cronbach’s α = 0.81); turning to religion (Cronbach’s α = 0.73).

#### 2.2.5. Couples’ dyadic adjustment dimensions

Couples’ dyadic adjustment dimensions were measured by using the Dyadic Adjustment Scale (DAS; Spanier, 1976; Gentili et al., 2002), which consists of 32 items divided into four subscales, namely dyadic consensus, affectional expression, dyadic cohesion, and dyadic satisfaction. Dyadic consensus measures the perception of agreement or disagreement with the partner on different issues such as finances, religion, household (13 items; Cronbach’s α = 0.90; e.g. “Aims, goals and things believed important”); affectional expression measures the perception of how affection, in terms of emotional and sexual life, is expressed within the couple (4 items; Cronbach’s α = 0.73; e.g., “sex relations”); dyadic cohesion measures the perception of the time spent in shared activities (5 items; Cronbach’s α = 0.86; e.g., “work together on a project”); dyadic satisfaction measures the perception of happiness or unhappiness in their relationship (10 items; Cronbach’s α = 0.94; e.g., “In general, how often do you think that things between you and your partner are going well?”). In the present study, Cronbach’s α values were satisfactory, i.e., dyadic consensus (Cronbach’s α = 0.71); affectional expression (Cronbach’s α = 0.77); dyadic cohesion (Cronbach’s α = 0.80); and dyadic satisfaction (Cronbach’s α = 0.79).

#### 2.2.6. State-anxiety and depression

Anxiety symptoms were measured by using the state scale from the State-Trait Anxiety Inventory (STAI-Y; Spielberger, 1972; Pedrabissi and Santinello, 1989), which consists of 20 items (e.g., “I am worried”) on a 4-point Likert scale ranging from one (not at all) to four (very much). The total score (Cronbach’s α = 0.91) ranges from 20 to 80 (Cronbach’s α = 0.91). State-anxiety scores were also converted into percentages and, according to the Italian validation study (Pedrabissi and Santinello, 1989), a score of 50.93 for female partners and 45.70 for male partners were considered to be the cut-off point in order to define the clinical cases. In the present study, Cronbach’s α value was satisfactory (Cronbach’s α = 0.84).

Depressive symptoms were measured by using the Edinburgh Depression Scale (EDS; Murray and Cox, 1990; Benvenuti et al., 1999) which consists of 10 items (e.g., “I have blamed myself unnecessarily when things went wrong”) on a 4-point Likert scale ranging from zero (not at all) to three (most of the time). The total score ranges from 0 to 30 (Cronbach’s α = 0.78). Depression scores were also converted into percentages and, according to the Italian validation study (Benvenuti et al., 1999), a score of 9.00 was considered to be the cut-off point to define the clinical cases. In the present study, Cronbach’s α value was satisfactory (Cronbach’s α = 0.82).

### 2.3. Data analysis

Descriptive statistics of study variables were computed and compared by gender (men/women) and by study group (PT-infertile couples/QTA-infertile couples). Firstly, in order to address *research question one* (*RQ1*), *t*-tests were carried out to compare mean scores of self-reported state-anxiety and depression. These study variables were also dichotomized into low and high levels referring to the clinical cut-off points reported by the Italian validation studies (see measure section), and frequencies and percentages of members of infertile couples reporting low and high (clinically relevant) levels of state-anxiety and depression were calculated and compared by study group across gender (cross-tabulations and *χ*^2^ analyses). Secondly, in order to address *research question two* (*RQ2*), *t*-tests were conducted, and differences in socio-demographics and infertility-related parameters (*RQ2.a*), in perceived levels of infertility-related stress dimensions (*RQ2.b*), in recourse to coping strategies (*RQ2.c*), and in perceived levels of couple’s dyadic adjustment dimensions (*RQ2.d*) were explored. Thirdly, a preliminary correlational analysis was undertaken to assess bivariate associations between all study variables by study group (Spearman’s correlations). Therefore, in order to address *research question three* (*RQ3*), the structural equation modelling (SEM) unconstrained approach put forward by Marsh et al. (2004) was carried out, among women and men respectively, to analyze the structural relationship between study variables (*RQ2*.a-b-c) and state-anxiety and depression by study group (PT-infertile couples/QTA-infertile couples). Finally, in order to address *research question four* (*RQ4*), further SEM models, were tested, among women and men respectively, to explore the potential moderating role of all socio-demographics and infertility-related parameters, coping strategies, and dyadic adjustment dimensions in the relationships between infertility-related stress dimensions and psychological health outcomes by study group (PT-infertile couples/QTA-infertile couples)(*RQ3*.a-b-c). The models’ fits were tested by using standard goodness-of-fit indices: Goodness-of-Fit (GFI > 0.90), Tucker–Lewis Index (TLI > 0.95), Comparative Fit Index (CFI > 0.95), and Root Mean Square Error of Approximation (RMSEA <0.08), Standardized Root Mean Square Residual (SRMR <0.08). Moreover, for analyzing and reporting moderation analysis, firstly the statistical significance of each moderating effect was examined, and then the R-sq values were explored to verify whether the inclusion of the interaction terms resulted in a statistically significant increase in the variance explained in the outcomes. All the statistical analyses were carried out by using SPSS (version 21) and AMOS tool (version 26).

## 3. Results

### 3.1. Research question one

Findings highlighted that members of QTA-infertile couples (both men and women) reported significantly lower levels of both state-anxiety and depression than members of PT-infertile couples (Table 1).

**Table 1:** State-anxiety and depression scores by gender and by study group.

	Women PT-infertile couples (*n* = 76)	Women QTA-infertile couples (*n* = 100)		Men PT-infertile couples (*n* = 76)	Men QTA-infertile couples (*n* = 100)	
**Psychological health outcomes**						
			*p* value			*p* value
State-anxiety	39.54 ± 11.05	36.43 ± 10.23	<0.001^a^	38.40 ± 8.92	35.54 ± 10.23	<0.001^a^
Low	35 (46.0)	62 (62.0)		38 (50.0)	67 (67.0)	
Clinically relevant	41 (54.0)	38 (38.0)	<0.001^b^	38 (50.0)	33 (33.0)	<0.001^b^
Depression	11.11 ± 4.67	8.22 ± 2.65	<0.001^a^	9.38 ± 2.99	7.52 ± 3.35	<0.001^a^
Low	37 (48.7)	65 (65.0)		44 (57.9)	71 (71.0)	
Clinically relevant	39 (51.3)	35 (35.0)	<0.001^b^	32 (42.1)	29 (29.0)	<0.001^b^

PT, persisting in medical treatments; QTA, quitting treatments and adopting. Differences are calculated by Student’s *t*-test (mean ± standard deviations)^a^ and Chi-square test [*N* (%)]^b^.

### 3.2. Research question two

Data revealed several statistically significant differences in study variables between women and men belonging to PT-infertile couples and QTA-infertile couples. In particular, considering socio-demographics and infertility-related parameters (*RQ2.a*; Table 2), data revealed that members of QTA-infertile couples (both men and women) reported significantly higher mean age and higher educational level (i.e., college) than members of PT-infertile couples. Moreover, women (but not men) belonging to QTA-infertile couples were also more likely to be employed than women belonging to PT-infertile couples. With respect to infertility-related parameters, PT-infertile couples reported significantly higher presence of Female Factor diagnosis and a greater number of failed ART treatments than QTA-infertile couples, while no significant differences were found with respect to duration of infertility.

**Table 2:** Socio-demographics and infertility-related parameters by gender and by study group.

Socio-demographics	Women PT (*n* = 76)	Women QTA (*n* = 100)	*p* value	Men PT (*n* = 76)	Men QTA (*n* = 100)	*p* value
Age years [*M* ± *SD*]	34.06 ± 3.22	35.95 ± 3.34	<0.01^a^	35.70 ± 3.57	37.34 ± 3.46	<0.001^a^
**Educational level [*N* (%)]**						
Upper Secondary School	28 (36.7%)	26 (26.0%)	<0.001^b^	32 (42.2%)	28 (28.0%)	<0.01^b^
College	48 (63.3%)	74 (74.0%)		44 (57.8%)	72 (72.0%)	
**Employment status [*N* (%)]**						
Unemployed	12 (15.8%)	10 (10.0%)	<0.001^b^	6 (8.0%)	7 (7.0%)	0.785^b^
Employed	64 (84.2%)	90 (90.0%)		70 (92.0%)	93 (93.0%)	
**Infertility-related parameters**	**PT-couples (*n* = 76)**	**QTA-couples (*n* = 100)**	***p* value**			
Duration of infertility [*M ± SD*]	3.65 ± 2.64	4.09 ± 2.87	0.512^a^			
Failed treatments [*M* + *DS*]	5.30 ± 2.43	3.09 ± 2.15	<0.001^a^			
**Type of diagnosis [*N* (%)]**						
Male factor	23 (30.9%)	30 (30.0%)	<0.01^b^			
Female factor	28 (36.8%)	31 (31.0%)				
Combined factor	15 (19.1%)	24 (24.0%)				
Unexplained	10 (13.2%)	15 (15.0%)				

PT, persisting in medical treatments; QTA, quitting treatments and adopting; M, mean; SD, standard deviation; N, number. Differences are calculated by Student’s *t-*test (mean ± standard deviations)^a^ and Chi-square test [*N* (%)]^b^.

Still responding to *RQ2*, considering perceived levels of infertility-related stress dimensions (*RQ2.b*; Table 3), data revealed that members of QTA-infertile couples (both men and women) reported significantly higher levels of perceived rejection of childfree lifestyle and lower levels of social concern and Couple’s relationship concern than members of PT-infertile couples. Moreover, women (but not men) belonging to QTA-infertile couples also reported significantly higher levels of perceived need for parenthood than women belonging to PT-infertile couples. Considering coping strategies (*RQ2.c*; Table 3), members of QTA-infertile couples (both men and women) displayed significantly greater recourse to social support and problem solving coping strategies and lower recourse to coping strategies centered on Avoiding than members of PT-infertile couples. Women (but not men) belonging to QTA-infertile couples also showed a significantly lower recourse to coping strategies centered on Turning to Religion than women belonging to PT-infertile couples. No significant differences emerged with respect to the recourse to positive attitude coping strategies.

**Table 3:** Infertility-related stress dimensions, adopted coping strategies, and perceived levels of couple’s dyadic adjustment by gender and by study group.

	Women PT-infertile couples (*n* = 76)	Women QTA-infertile couples (*n* = 100)	*p* value	Men PT-infertile couples (*n* = 76)	Men QTA-infertile couples (*n* = 100)	*p* value
	Mean ± SD	Mean ± SD		Mean ± SD	Mean ± SD	
**Infertility-related stress dimensions**						
Social concern	28.45 ± 10.48	23.04 ± 11.77	<0.001	26.38 ± 9.59	20.47 ± 9.93	<0.001
Need for parenthood	27.12 ± 6.38	29.48 ± 7.22	<0.001	26.48 ± 5.32	26.99 ± 6.37	0.364
Rejection of childfree lifestyle	23.76 ± 9.24	26.85 ± 9.55	<0.001	22.47 ± 8.48	25.75 ± 9.75	<0.001
Couple’s relationship concern	13.75 ± 5.42	10.66 ± 5.28	<0.001	12.89 ± 6.20	10.02 ± 5.18	<0.001
**Coping strategies**						
Social support	24.51 ± 4.86	28.74 ± 6.33	<0.001	22.62 ± 5.17	26.44 ± 6.50	<0.001
Avoiding	25.38 ± 6.13	22.08 ± 5.26	<0.001	27.23 ± 6.09	23.35 ± 5.87	<0.001
Positive attitude	29.04 ± 6.52	29.85 ± 6.23	0.473	30.04 ± 5.82	30.62 ± 5.25	0.574
Problem solving	23.15 ± 4.84	29.07 ± 6.23	<0.001	22.26 ± 6.24	27.35 ± 6.73	<0.001
Turning to religion	26.15 ± 4.45	24.92 ± 4.82	<0.001	24.34 ± 4.64	23.96 ± 5.03	0.292
**Dyadic adjustment dimensions**						
Dyadic consensus	50.29 ± 8.01	53.67 ± 7.85	<0.001	51.53 ± 6.57	54.35 ± 6.98	<0.001
Dyadic cohesion	16.96 ± 3.82	18.95 ± 4.04	<0.01	17.63 ± 3.65	17.46 ± 3.78	0.438
Dyadic satisfaction	30.83 ± 6.26	33.82 ± 5.02	<0.001	31.35 ± 5.69	33.06 ± 4.62	<0.01
Affectional expression	9.96 ± 1.84	11.67 ± 2.04	<0.01	10.35 ± 1.70	10.78 ± 1.94	0.563

PT, persisting in medical treatments; QTA, quitting treatments and adopting; M, mean; SD, standard deviation. Differences are calculated by Student’s *t* test.

Finally, considering dyadic adjustment dimensions (*RQ2.d*; Table 3), data showed that members of QTA-infertile couples (both men and women) reported significantly higher levels of perceived dyadic consensus and dyadic satisfaction than members of PT-infertile couples. Furthermore, women (but not men) belonging to QTA-infertile couples also reported significantly higher levels of perceived dyadic cohesion and affectional expression than women belonging to PT-infertile couples.

### 3.3. Research question three

Preliminary to SEM models, Spearman’s correlations were conducted between study variables and findings are reported in Table 4.

**Table 4:** Bivariate correlations between study variables in PT-infertile couples and in QTA-infertile couples.

	1	2	3	4	5	6	7	8	9	10	11	12	13	14	15	16	17	18	19	20	21	22	23	24	25
1. Gender	1	0.26^*^	−0.12	0.39^**^	−0.10	−0.11	0.12	−0.08	0.11	0.10	0.39^**^	0.10	−0.11	0.11	−0.10	0.23^*^	0.02	−0.10	−0.11	−0.40^**^	−0.13	−0.11	−0.12	0.11	0.12
2. Age	0.37^**^	1	0.25^*^	0.38^**^	0.39^**^	0.27^*^	0.12	0.25^*^	0.08	0.12	0.12	0.10	0.45^**^	0.27^*^	−0.26^*^	−0.11	−0.11	0.04	0.48^**^	−0.47^**^	−0.42^**^	−0.11	−0.10	0.44^**^	0.37^**^
3. Educational Level	0.11	0.34^*^	1	0.37^**^	0.09	−0.07	0.12	0.14	0.09	0.11	−0.13	−0.09	−0.13	−0.24^*^	0.27^*^	−0.09	0.11	0.33^**^	0.09	0.12	0.14	0.08	0.11	−0.33^**^	−0.11
4. Employment Status	0.42^**^	0.33^*^	0.36^**^	1	0.12	0.11	0.24^*^	0.12	0.11	−0.11	−0.10	0.12	0.14	−0.08	0.23^*^	−0.11	0.12	0.28^*^	0.11	0.23^*^	0.10	0.27^*^	0.13	−0.41^**^	−0.10
5. Duration of Infertility	−0.11	0.44^**^	0.13	0.08	1	0.47^**^	0.12	0.30^*^	0.12	0.11	0.32^**^	0.07	0.12	0.26^*^	−0.11	0.10	−0.09	−0.12	0.07	−0.24^*^	−0.10	−0.15	−0.36^**^	0.45^**^	0.33^**^
6. Failed Treatments	−0.10	0.39^**^	0.10	−0.06	0.26^*^	1	0.13	0.10	0.11	0.13	0.36^**^	0.13	0.08	0.12	−0.08	0.38^**^	−0.25^*^	−0.12	0.24^*^	−0.12	−0.25^*^	−0.12	−0.35^**^	0.46^**^	0.41^**^
7. Male Factor Diagnosis	0.06	0.13	0.11	0.44^**^	0.13	0.06	1	−0.08	−0.07	−0.11	0.37^**^	0.10	0.13	0.15	−0.12	0.44^**^	0.11	0.14	−0.10	0.13	−0.12	−0.06	0.25^*^	0.36^**^	0.31^*^
8. Female Factor Diagnosis	0.06	0.40^**^	0.10	0.11	0.37^**^	0.25^*^	−0.06	1	−0.07	−0.04	0.28^*^	0.23^*^	0.13	0.11	0.13	0.11	0.24^*^	0.13	0.25^*^	0.11	0.23^*^	−0.12	−0.10	0.40^**^	38^**^
9. Combined Factor Diagnosis	0.10	0.06	0.11	0.14	0.08	0.07	−0.06	−0.09	1	−0.08	0.25^*^	0.12	0.24^*^	0.12	0.11	0.26^*^	−0.04	−0.13	0.24^*^	−0.13	−0.12	−0.27^*^	−0.12	0.39^**^	0.27^*^
10. Unexplained Diagnosis	0.12	0.10	0.22^*^	0.12	−0.11	0.12	−0.26^*^	−0.11	−0.09	1	0.24^*^	0.12	0.14	0.26^*^	−0.09	0.38^**^	−0.10	−0.07	0.12	0.34^*^	−0.08	−0.11	0.13	0.36^**^	31^*^
11. Social Concern	0.30^*^	0.12	−0.10	−0.09	0.24^*^	0.30^*^	−0.12	0.12	0.09	0.10	1	0.11	0.05	0.47^**^	−0.12	0.25^*^	−0.11	−0.10	0.05	−0.50^**^	−0.46^**^	−0.41^**^	−0.38^**^	0.40^**^	0.42^**^
12. Need for Parenthood	0.11	0.40^**^	0.24^*^	0.10	0.11	0.09	0.11	0.25^*^	0.09	0.10	0.12	1	0.42^**^	0.53^**^	0.04	−0.11	0.10	0.11	0.44^**^	−0.37^**^	0.11	0.13	−0.44^**^	−0.08	0.27^*^
13. Rejection of Childfree Lifestyle	0.06	0.11	0.12	0.09	0.11	0.06	0.24^*^	0.26^*^	0.25^*^	0.11	0.11	0.41^**^	1	0.13	−0.45^**^	−0.13	0.07	0.12	0.34^**^	0.08	0.35^**^	−0.08	−0.09	−0.05	0.11
14. Couple’s Relationship Concern	0.11	0.07	0.12	0.09	0.12	0.11	0.26^*^	0.12	−0.08	0.11	0.46^**^	0.12	0.11	1	−0.44^**^	0.33^**^	0.07	0.10	0.06	−0.47^**^	−0.50^**^	−0.32^*^	−0.48^**^	0.44^**^	0.40^**^
15. Social Support coping	−0.29^*^	0.10	0.26^*^	0.12	0.07	0.11	−0.11	0.29^*^	−0.09	−0.12	0.05	0.48^**^	0.40^**^	0.44^**^	1	−0.50^**^	0.11	0.10	0.12	−0.06	−0.12	0.27^*^	0.11	0.13	0.13
16. Avoiding coping	0.26^*^	−0.11	−0.06	−0.10	0.11	0.08	0.26^*^	0.08	0.12	0.11	0.09	−0.44^**^	−0.07	0.12	0.10	1	−0.38^**^	−0.47^**^	0.10	0.08	−0.49^**^	−0.10	0.09	0.11	0.34^*^
17. Positive Attitude coping	0.11	0.25^*^	0.11	0.13	−0.07	−0.04	−0.11	0.25^*^	0.09	−0.12	0.09	0.22	−0.07	−0.11	0.47^**^	−0.30^*^	1	0.10	0.13	0.27^*^	0.10	0.09	0.42^**^	−0.40^**^	0.11
18. Problem Solving coping	0.09	0.30^*^	0.26^*^	0.10	−0.08	−0.07	0.24^*^	0.27^*^	0.11	0.14	0.12	0.58^**^	0.45^**^	0.13	0.36^**^	0.09	0.32^*^	1	0.09	0.11	0.07	0.44^**^	0.11	0.10	0.12
19. Turning to Religion coping	0.02	0.11	−0.08	−0.04	0.12	0.10	−0.09	0.13	0.24^*^	0.10	−0.05	0.10	0.07	0.08	0.09	0.02	0.07	0.08	1	0.12	0.05	0.43^**^	0.06	−0.40^**^	0.11
20. Dyadic Consensus	−0.10	0.11	0.09	0.12	−0.08	−0.11	0.26^*^	0.28^*^	0.10	0.11	−0.24^*^	0.09	0.44^**^	0.07	0.30^*^	−0.26^*^	0.39^**^	0.40^**^	0.11	1	0.42^**^	0.60^**^	0.49^**^	−0.47^**^	−0.29^*^
21. Dyadic Satisfaction	0.02	0.11	0.09	0.10	−0.10	−0.12	−0.10	0.26^*^	0.27^*^	0.09	−0.31^*^	−0.07	0.02	−0.28^*^	0.09	−0.29^*^	0.08	0.11	0.16	0.57^**^	1	0.48^**^	0.49^**^	−0.44^**^	−0.39^**^
22. Dyadic Cohesion	−0.08	0.12	0.08	0.14	−0.06	0.08	0.12	0.27^*^	0.11	0.09	−0.24^*^	−0.12	0.08	−0.06	0.11	−0.08	0.30^*^	0.09	−0.05	0.66^**^	0.42^**^	1	0.47^**^	−0.52^**^	−0.09
23. Affectional Expression	0.05	−0.09	0.08	0.12	−0.08	−0.13	0.26^*^	0.10	0.25^*^	0.08	−0.12	−0.11	−0.09	0.10	0.08	−0.07	0.04	0.08	−0.06	0.50^**^	0.38^**^	0.56^**^	1	−0.52^**^	−0.51^**^
24. State-Anxiety	0.03	0.36^**^	−0.26^*^	−11	0.28^*^	0.39^**^	0.24^*^	0.26^*^	0.27^*^	0.24^*^	0.49^**^	0.25^*^	0.30^*^	0.25^*^	−0.39^**^	0.23^*^	−0.40^**^	−0.47^**^	0.24^*^	−0.49^**^	0.05	−0.48^**^	−0.07	1	0.51^**^
25. Depression	0.12	0.30^*^	−0.26^*^	−0.23^*^	0.24^*^	0.39^**^	0.44^**^	0.46^**^	0.23^*^	0.11	0.09	0.38^**^	0.10	0.49^**^	0.10	0.46^**^	−0.08	−0.38^**^	0.07	−0.59^**^	−0.11	−0.08	−0.09	0.46^**^	1

PT, persisting in medical treatments; QTA, quitting treatments and adopting. PT-infertile couples correlations are reported above the diagonal, QTA-infertile couples correlations are reported below the diagonal. *p < 0.05; **p < 0.01.

**Figure 1:**
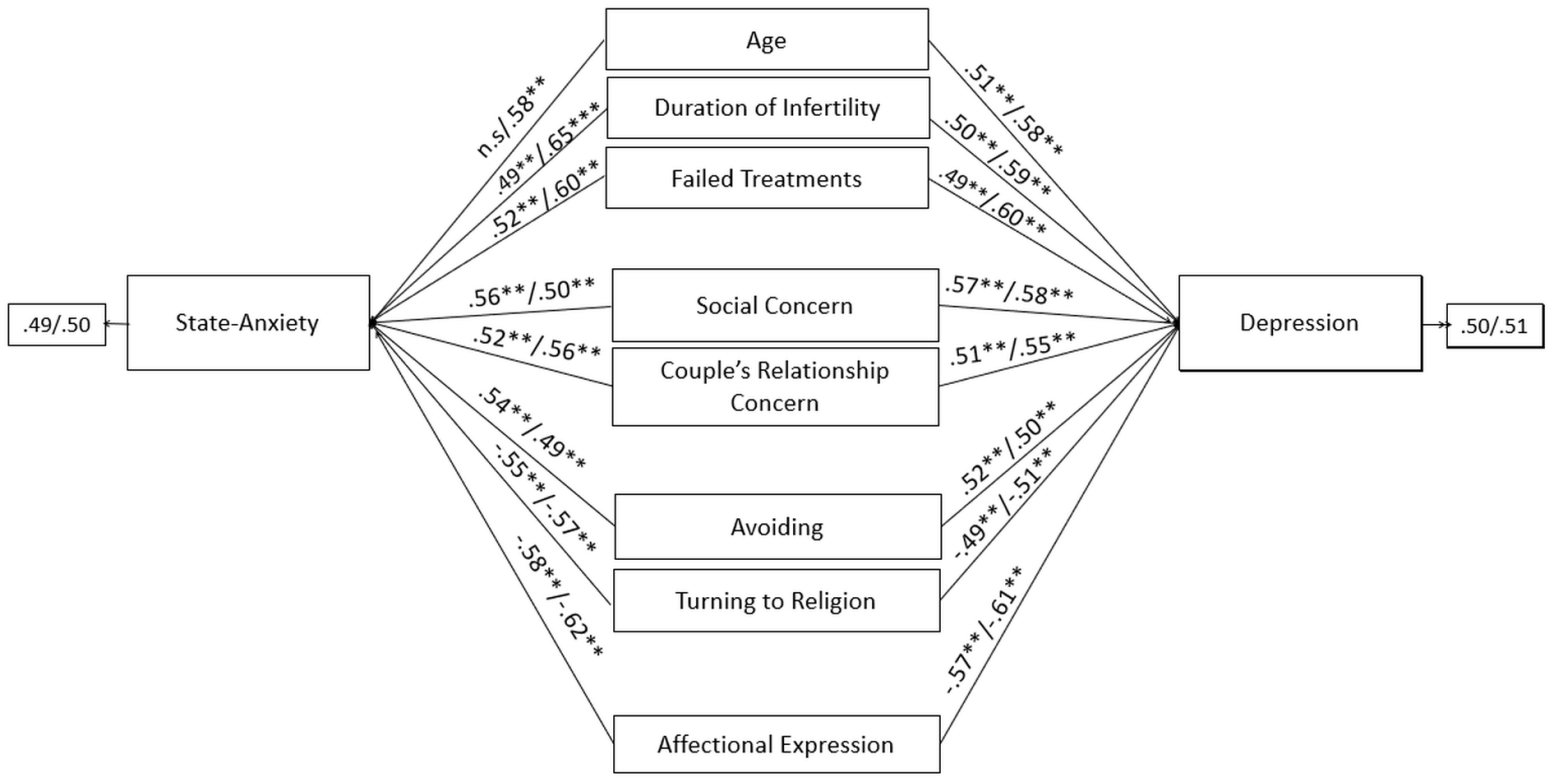
Predictors of state-anxiety and depression in male and female members of PT-infertile couples: path models. Standardized regression coefficients are provided along the paths. The first coefficient in each path refers to men, whereas the second refers to women. ^∗^*p* < 0.05, ^∗∗^*p* < 0.01, ^∗∗∗^*p* < 0.001.

Therefore, responding to *RQ3*, specific SEM models according to the two study groups were found. The final predictive models of psychological health outcomes for men (state-anxiety: GFI = 0.968, TLI = 0.971, CFI = 0.975, RMSEA = 0.055, SRMR = 0.063; depression: GFI = 0.979, TLI = 0.967, CFI = 0.980, RMSEA = 0.057, SRMR = 0.058) and women (state-anxiety: GFI = 0.971, TLI = 0.974, CFI = 0.973, RMSEA = 0.055, SRMR = 0.062; depression: GFI = 0.977, TLI = 0.968, CFI = 0.981, RMSEA = 0.054, SRMR = 0.055) belonging to couples persisting in treatments (PT) showed adequate goodness of fit. Overall, both for male and female partners, the following predictors of state-anxiety/depression were found: age, duration of infertility, number of failed treatments, the infertility-related stress dimensions of social concern and couple’s relationship concern, avoiding and turning to religion coping strategies, and the couple’s adjustment dimension of affectional expression. Data did not reveal substantial gender differences within the members of PT couples, as age represented a significant predictor of state-anxiety only among female partners, but it was a significant predictor of depression among both members of PT couples (Figure 1).

**Figure 2:**
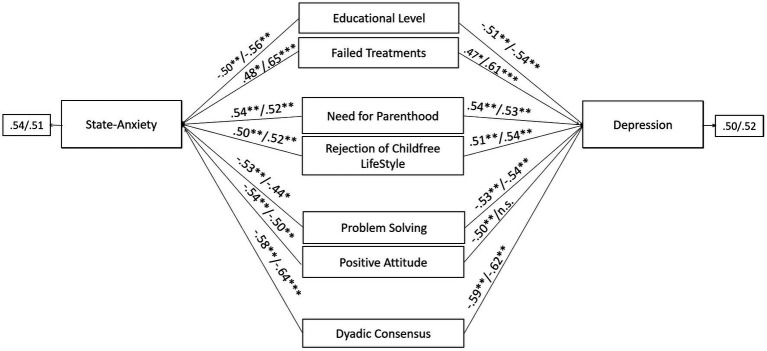
Predictors of state-anxiety and depression in male and female members of QTA-infertile couples: path models. Standardized regression coefficients are provided along the paths. The first coefficient in each path refers to men, whereas the second refers to women. ^∗^*p* < 0.05, ^∗∗^*p* < 0.01, ^∗∗∗^*p* < 0.001.

The final predictive models of psychological health outcomes for men (state-anxiety: GFI = 0.974, TLI = 0.974, CFI = 0.977, RMSEA = 0.056, SRMR = 0.053; depression: GFI = 0.973, TLI = 0.963, CFI = 0.967, RMSEA = 0.056, SRMR = 0.058) and women (state-anxiety: GFI = 0.976, TLI = 0.978, CFI = 0.978, RMSEA = 0.055, SRMR = 0.052; depression: GFI = 0.974, TLI = 0.970, CFI = 0.972, RMSEA = 0.054, SRMR = 0.051) belonging to couples opting for quitting treatment and adopting (QTA) showed adequate goodness of fit. Overall, both for male and female partners, the following predictors of state-anxiety/depression were found: educational level, number of failed treatments, the infertility-related stress dimensions of need for parenthood and rejection of childfree lifestyle, problem solving and positive attitude coping strategies, and the couple’s adjustment dimension of dyadic consensus. Data did not reveal substantial gender differences within the members of QTA couples, as positive attitude represented a significant predictor of depression only among female partners, but it was a significant predictor of state-anxiety among both members of QTA couples (Figure 2).

### 3.4. Research question four

Specific moderators of the relationship between infertility-related stress dimensions and perceived levels of state-anxiety and depression were found according to the study group. No gender specificities were found within PT and QTA couples, respectively.

With respect to male and female partners of PT-infertile couples, SEM models highlighted several statistically significant moderating effects that will be reported as follows.

**Figure 3:**
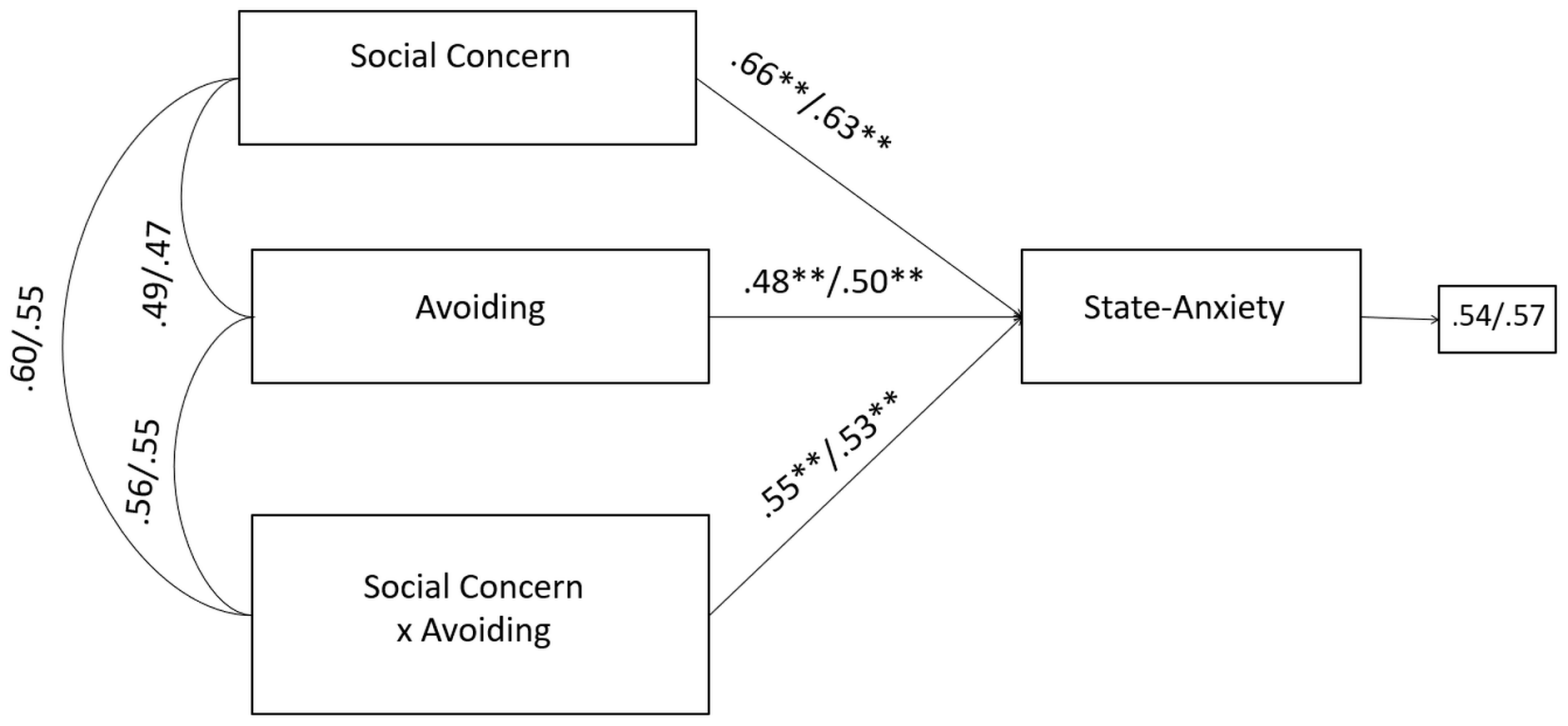
A moderate model of social concern and state-anxiety through avoiding coping in male and female members of PT-infertile couples. Standardized regression coefficients are provided along the paths. The first coefficient in each path refers to men, whereas the second refers to women. ^∗^*p* < 0.05, ^∗∗^*p* < 0.01, ^∗∗∗^*p* < 0.001.

Firstly, the negative effects of the infertility-related stressor of social concern on state-anxiety were significantly exacerbated by avoiding coping strategy. Without the inclusion of the moderating effect (social concern × avoiding coping), the R-sq value for state-anxiety was 0.378 for male and 0.385 for female partners. This shows that 37.8 and 38.5% change in state-anxiety was accounted by social concern and avoiding. With the inclusion of the interaction term, the R-sq revealed a statistically significant increase of 5.4 and 10.6%, respectively for male and female, reaching the values of 43.2% for male and 49.1% for female in the variance explained in state-anxiety (Figure 3).

**Figure 4:**
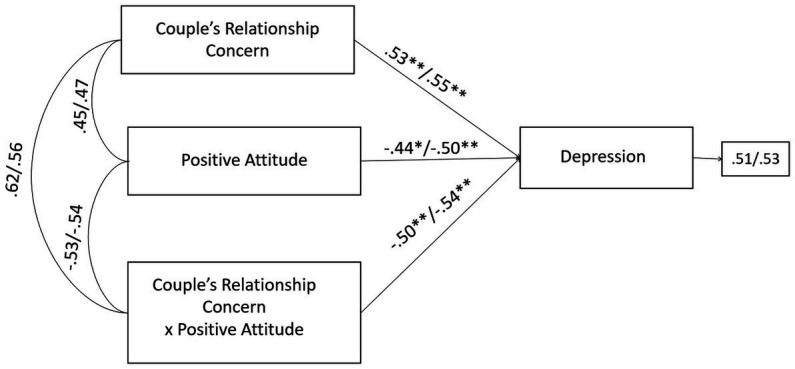
A moderate model of couple’s relationship concern and depression through positive attitude coping in male and female members of PT-infertile couples. Standardized regression coefficients are provided along the paths. The first coefficient in each path refers to men, whereas the second refers to women. ^∗^*p* < 0.05, ^∗∗^*p* < 0.01, ^∗∗∗^*p* < 0.001.

Furthermore, the negative effects of the infertility-related stressor of couple’s relationship concern on depression were significantly buffered by positive attitude coping strategy. Without the inclusion of the moderating effect (couple’s relationship concern × positive attitude coping), the R-sq value for depression was 0.354 for male and 0.314 for female partners. This shows that 35.4 and 31.4% change in depression was accounted by couple’s relationship concern and positive attitude coping. With the inclusion of the interaction term, the R-sq revealed a statistically significant increase of 6.1 and 8.4%, respectively for male and female, reaching the values of 41.5% for male and 39.8% for female in the variance explained in depression (Figure 4).

**Figure 5:**
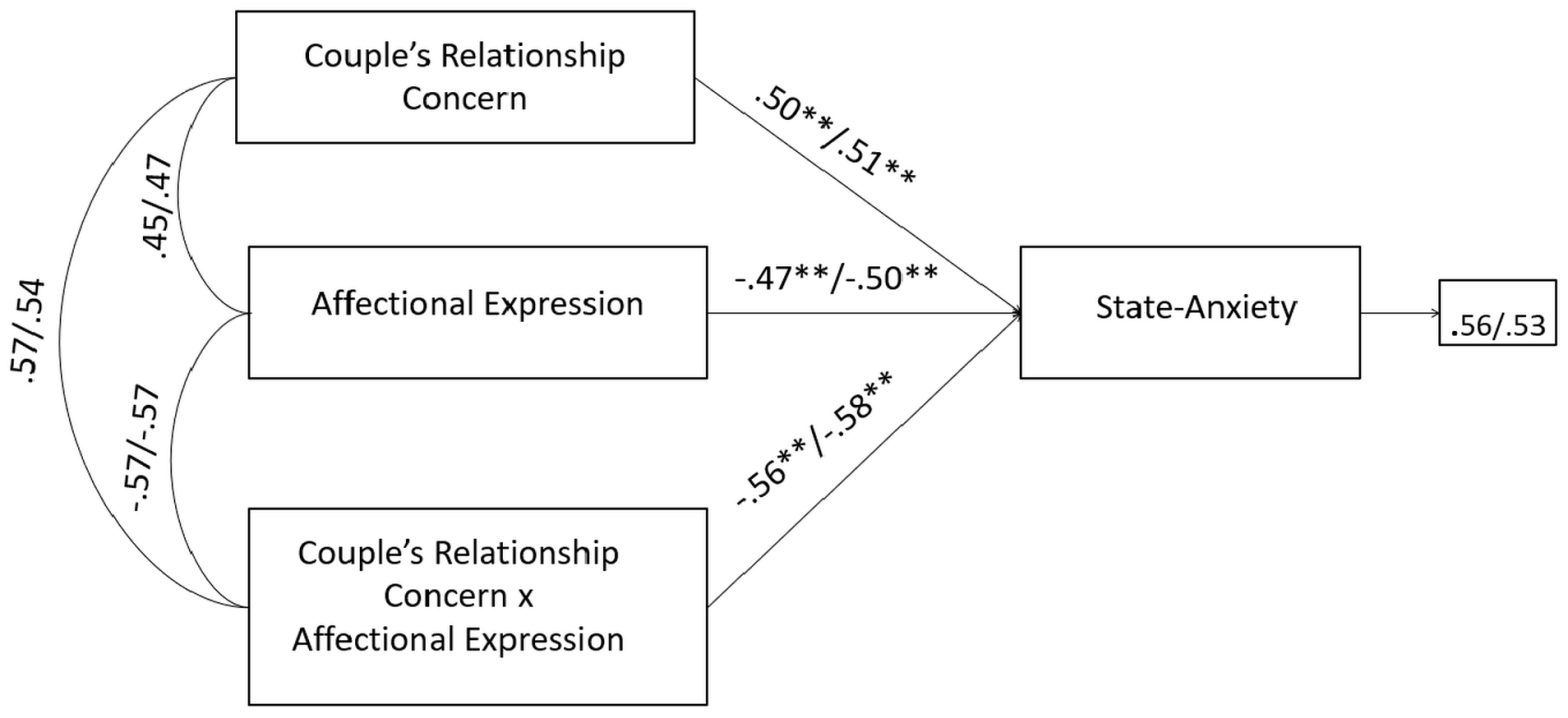
A moderate model of couple’s relationship concern and state-anxiety through affectional expression in male and female members of PT-infertile couples. Standardized regression coefficients are provided along the paths. The first coefficient in each path refers to men, whereas the second refers to women. ^∗^*p* < 0.05, ^∗∗^*p* < 0.01, ^∗∗∗^*p* < 0.001.

Finally, the negative effects of the infertility-related stressor of couple’s relationship concern on state-anxiety were significantly buffered by the couple’s adjustment dimension of affectional expression. Without the inclusion of the moderating effect (couple’s relationship concern × affectional expression), the R-sq value for state-anxiety was 0.438 for male and 0.366 for female partners. This shows that 43.8 and 36.6% change in state-anxiety was accounted by couple’s relationship concern and affectional expression. With the inclusion of the interaction term, the R-sq revealed a statistically significant increase of 7.6 and 5.9%, respectively for male and female, reaching the values of 51.4% for male and 42.5% for female in the variance explained in state-anxiety (Figure 5). No further significant moderating effects were found for PT-study group.

**Figure 6:**
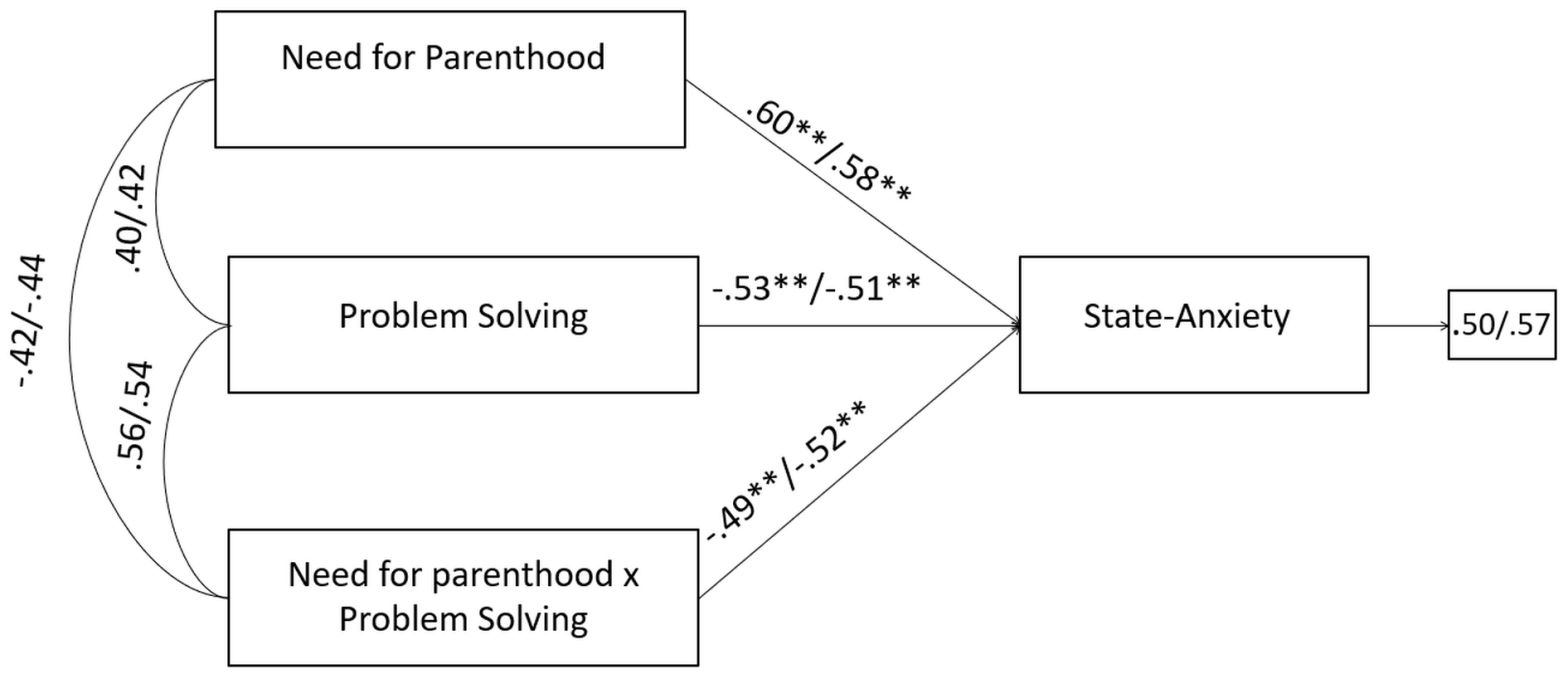
A moderate model of need for parenthood and state-anxiety through problem solving in male and female members of QTA-infertile couples. Standardized regression coefficients are provided along the paths. The first coefficient in each path refers to men, whereas the second refers to women. ^∗^*p* < 0.05, ^∗∗^*p* < 0.01, ^∗∗∗^*p* < 0.001.

Correspondingly, with respect to male and female partners of QTA-infertile couples, SEM models highlighted, firstly, that the negative effects of the infertility-related stressor of need for parenthood on state-anxiety were significantly buffered by problem solving coping strategy without the inclusion of the moderating effect (need for parenthood × problem solving coping), the R-sq value for state-anxiety was 0.344 for male and 0.399 for female patients. This shows that 34.4 and 39.9% change in state-anxiety was accounted by need for parenthood and problem solving coping. With the inclusion of the interaction term, the R-sq revealed a statistically significant increase of 5.8 and 5.2%, respectively for male and female, reaching the values of 40.2% for male and 45.1% for female in the variance explained in state-anxiety (Figure 6).

**Figure 7:**
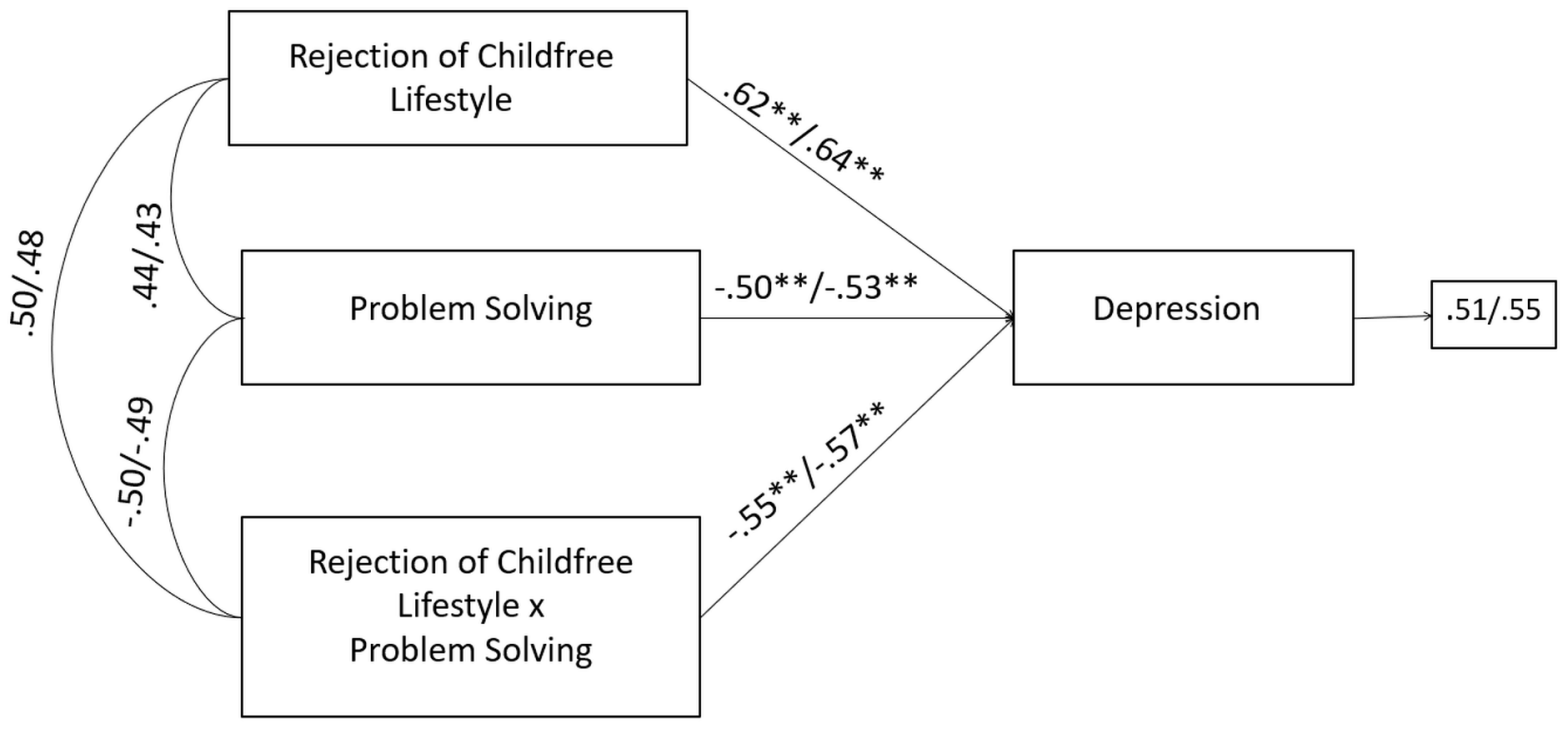
A moderate model of rejection of childfree lifestyle and depression through problem solving in male and female members of QTA-infertile couples. Standardized regression coefficients are provided along the paths. The first coefficient in each path refers to men, whereas the second refers to women. ^∗^*p* < 0.05, ^∗∗^*p* < 0.01, ^∗∗∗^*p* < 0.001.

Moreover, the negative effects of the infertility-related stressor of rejection of childfree lifestyle on depression were significantly buffered by problem solving coping strategy. Without the inclusion of the moderating effect (rejection of childfree lifestyle × problem solving), the R-sq value for depression was 0.343 for male and 0.359 for female partners. This shows that 34.3 and 35.9% change in state-anxiety was accounted by rejection of childfree lifestyle and problem solving. With the inclusion of the interaction term, the R-sq revealed a statistically significant increase of 7.9 and 5.8%, respectively for male and female, reaching the values of 42.2% for male and 41.7% for female in the variance explained in depression (Figure 7).

**Figure 8:**
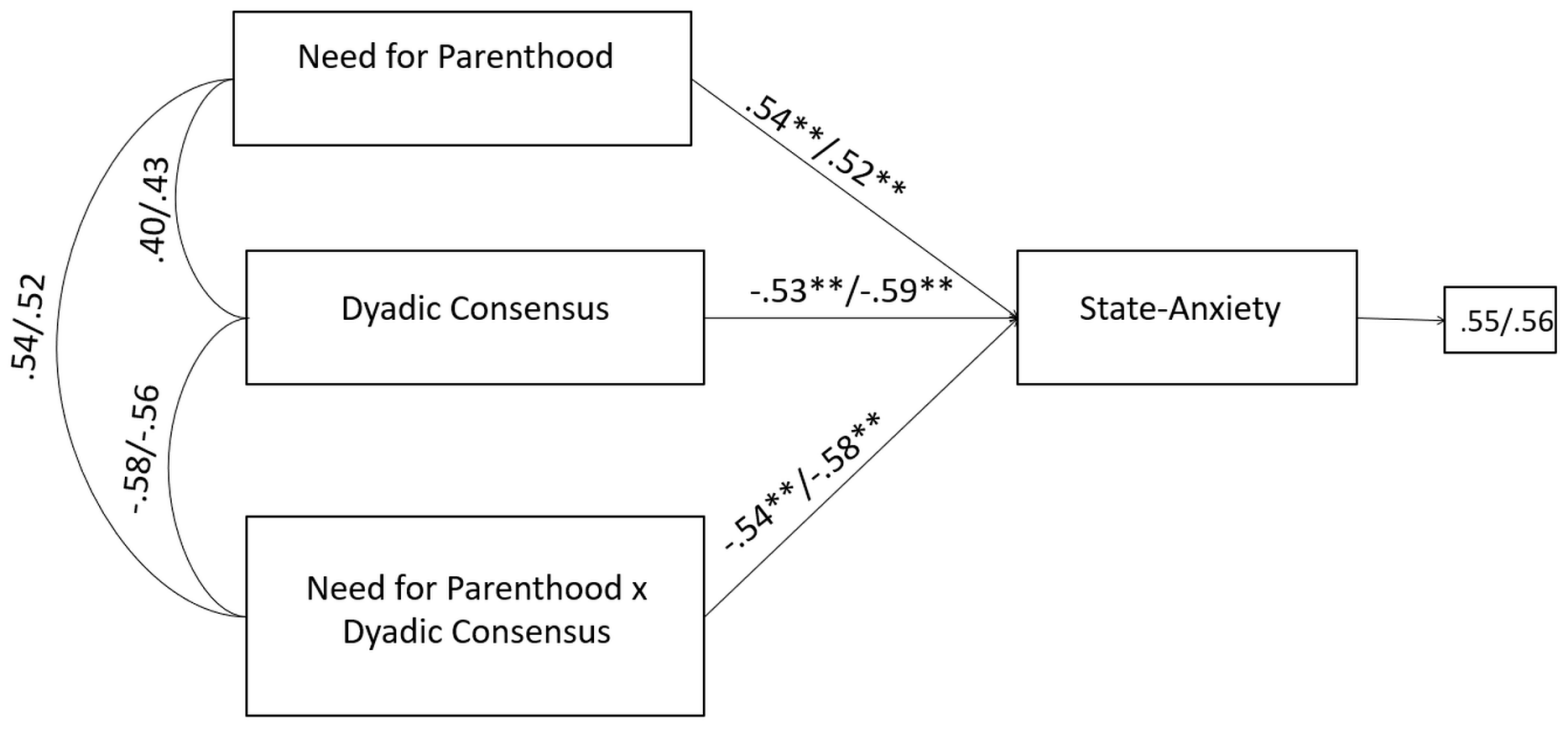
A moderate model of need for parenthood and state-anxiety through dyadic consensus in male and female members of QTA-infertile couples. Standardized regression coefficients are provided along the paths. The first coefficient in each path refers to men, whereas the second refers to women. ^∗^*p* < 0.05, ^∗∗^*p* < 0.01, ^∗∗∗^*p* < 0.001.

Finally, the negative effects of the infertility-related stressor of need for parenthood on state-anxiety were significantly buffered by the couple’s adjustment dimension of dyadic consensus. Without the inclusion of the moderating effect (need for parenthood × dyadic consensus), the R-sq value for state-anxiety was 0.365 for male and 0.340 for female partners. This shows that 36.5 and 34.0% change in state-anxiety was accounted by need for parenthood and dyadic consensus. With the inclusion of the interaction term, the R-sq revealed a statistically significant increase of 6.7 and 6.8%, respectively for male and female, reaching the values of 43.2% for male and 40.8% for female in the variance explained in state-anxiety (Figure 8). No further significant moderating effects were found for QTA-study group.

## 4. Discussion

Basing on a transactional and multidimensional approach to infertility-related stress and health, the present study aimed at exploring individual predictors (socio-demographics; coping strategies) and situational (infertility-related parameters and stressors; couple’s dyadic adjustment dimensions) predictors of state-anxiety and depression in infertile couples pursuing treatments (PT) or quitting them and opting for adoption (QTA). However, the first research step made was towards the exploration of any difference between male and female partners belonging to PT- and QTA-infertile couples.

Therefore, firstly, data revealed that both members of PT-infertile couples reported significantly higher levels of state-anxiety and depression than QTA-infertile couples (*RQ1*), thus highlighting higher psychological suffering among couples still undergoing ART treatments in comparison to the couples who reached the shared decision to quit treatments and opt for adoption to achieve parenthood. Moreover, when considering the rates of clinically relevant levels of state-anxiety and depression reported by members of PT-infertile couples (i.e., state-anxiety: 54% of women and 50% of men; depression: 51.3% of women and 42.1% of men) they also reported higher psychological disease than a comparable sample of Italian infertile couples (i.e., clinically relevant levels of state-anxiety: 27.2% of women and 34% of men; clinically relevant levels of depression: 53.6% of women and 40% of men; Zurlo et al., 2020a).

These findings are in line with research highlighting higher psychological disease in infertile couples undergoing treatments, when compared with both adoptive and fertile couples (Galhardo et al., 2011), as well as with studies emphasizing a great risk of depression and notable levels of anxiety reported by patients before and during infertility treatments (Massarotti et al., 2019), with exacerbation of mental health risk linked to treatment failure and longer duration of treatments (Gdańska et al., 2017; Zurlo et al., 2018).

However, in line with qualitative studies (Bartholet, 1993; Daniluk and Hurtig-Mitchell, 2003), we have hypothesized these data may also reflect the possibility that the adoptive path can help to ameliorate the negative impact of infertility, fostering the opportunity of healing the hurt and the grief of being infertile, as well as of affording couples the “potential for transformation and rebirth.”

Nonetheless, despite members of QTA-infertile couples displaying significantly better psychological health than PT-infertile couples, 38% of women and 33% of men reported clinically relevant levels of state-anxiety, while 35% of women and 29% of men reported clinically relevant levels of depression. This implies the necessity to carefully consider that there is no lack of burden and psychological costs within the adoptive choice. Indeed, QTA-infertile couples still need to be supported in acknowledging and elaborating on the anger, the feeling of frustration, and grief for the several losses associated with their infertility (e.g., the inability to produce a child sharing genetic/social histories; the experience of pregnancy; failure of treatments) to move forward and consider their alternative options. These latter still potentially entail perceived powerlessness (e.g., the success of adopting procedure) and new challenges linked to parenting. Therefore, overall these data fully endorse the need to investigate and compare predictors of psychological health to identify risks and resources in order to develop evidence-based tailored interventions for PT-infertile couples and QTA-infertile couples, respectively.

In this direction, considering socio-demographics and infertility-related parameters (*RQ2.a*), data firstly revealed that members of QTA-infertile couples (mainly women) reported significantly higher mean age. Considering that older age also means a lower chance of ART treatment success (Liu et al., 2011; Human Fertilization and Embryology Authority [HFEA], 2018, 2019) it can be also hypothesized this factor may represent a meaningful drive supporting couples’ choice to consider adoption. On the other side, members of QTA-couples also possessed – to a greater extent than PT-couples – specific individual resources (i.e., higher educational level and employment rates) that have been well-recognized in literature as fostering infertile patients’ possibility to draw and rely on other aspects to affirm/preserve own identity (Ramezanzadeh et al., 2004; Noorbala et al., 2009; Alhassan et al., 2014; Zurlo et al., 2018) and that we hypothesize can also potentially serve to overwhelm the negative attitude towards adoption (e.g., concerns of blood-tie preservation, racial prejudices, keeping infertility as a secret; Bharadwaj, 2003; Goldberg et al., 2009; Rolnick and Pearson, 2017).

Furthermore, data also suggested specificities in medical parameters by study group, indicating that, despite there being no difference in the duration of infertility, QTA-infertile couples have experienced a significantly lower number of failed treatments (*M* = 3.09, *SD* = 2.15) than PT-infertile couples (*M* = 5.30, *SD* = 2.43). This seems to suggest that the processes of reflection and elaboration, leading to the turning point disclosing the decision-making process may begin earlier in members of QTA-infertile couples, ever since the first years of infertility and failure experiences. However, this may be also linked to the evidence that QTA-infertile couples were more likely diagnosed with combined factor, which directly involves both members of couples in infertility experience, treatment and choices on it. Nonetheless, the lower number of failed treatments could be also the result of a lower number of treatments they actually underwent, especially if they had severe diagnoses and/or they were counseled by the physician about their poor chance of success. Differently, our data also revealed that PT-infertile couples were more frequently diagnosed with female factor. We hypothesize that since the medical treatment mainly involves and challenges women’s bodies, when the diagnosis lies with them, the choice of quitting treatments can be more difficult to achieve than when the diagnosis involves the men or the couple, due to the potentially more intense sense of shame, guilt, and powerlessness.

With respect to perceived infertility-related stress dimensions (*RQ2.b*), coping strategies (*RQ2.c*), and perceived dyadic adjustment dimensions (*RQ2.d*), data showed that members of QTA-infertile couples perceived significantly higher levels of stress related to need for parenthood and rejection of childfree lifestyle. This may suggest that the inability of imagining a life without children and the wish to achieve parenthood embody the most challenging infertility-related issues among QTA-infertile couples, potentially representing the key drive fostering the adoptive choice and allowing to overwhelm the concerns linked, for example, to the bloodline preservation (Miall, 1987; Petropanagos, 2010).

In the same direction, members of QTA-infertile couples recurred to a greater extent to active coping strategies (problem solving and seeking social support) so displaying a more engaged adjustment profile than members of PT-infertile couples.

Finally, members of QTA-infertile couples also perceived significantly higher levels of dyadic adjustment (mainly in terms of dyadic satisfaction and consensus) suggesting that opting for family-building alternatives, such as adoption, may be linked to a satisfactory couple balance (Thorn, 2010).

Differently, PT-infertile couples perceived significantly higher levels of stress related to social concern and couple’s relationship concern. This may suggest that the perception of social stigma concerning infertility – also determined in some contexts by the perceived negative views by society, family, and friends – and the impact of infertility experiences as damaging the couple’s balance embody the most challenging infertility-related issues among PT-infertile couples, potentially representing the key drive for persisting in pursuing medical treatments, even after cumulative unsuccessful experiences.

Noteworthy, data showed that members of PT-infertile couples also reported statistically relevant greater recourse to passive coping strategies (i.e., avoiding and turning to religion coping strategies), namely an adjustment profile characterized by denying, withdrawing, and delegating control to others. We hypothesize the above-mentioned profile should be carefully considered to reflect upon the behavior to persist in treatments, which – in some cases – could also result from the predominantly adoption of this set of coping strategies, rather than being an active choice.

Finally, PT-infertile couples reported significantly lower levels of perceived dyadic adjustment. We believe this may be partly due to the difficulties and challenges faced by the couples undertaking prolonged treatments and dealing with repeated failures. Indeed, in many cases, the infertility path may impair intimacy and sexual life, significantly compromising the enjoyment of the relationship (Samplaski and Nangia, 2015).

Findings from SEM models allowed the identification of specific risks and resources (main and moderating effects; *RQ3* and *RQ4*) for psychological health outcomes among male and female partners of PT and QTA couples, also highlighting further substantial and significant differences by study group. Noteworthy, our findings did not reveal substantial gender differences in the predictors of psychological health outcomes within each study group, so that we can gain implications and provide tailored recommendations - based on our findings - that could be used to develop interventions among both members of couples belonging to PT and QTA couples, respectively.

Specifically, when defining interventions targeting male and female members of PT-infertile couples, our data suggested specific risks and resources that clinicians should take into account, i.e., higher age (main negative effect), increasing duration of infertility and number of treatment failures, the recourse to avoiding coping strategy (both main and moderating negative effects), and the perception of high levels of social concern and couple’s relationship concern represented significant risk factors, while the recourse to positive attitude (moderating buffering effects) and to turning to religion coping strategy, and the perception of couples’ affectional expression (both main and moderating buffering effects) represented significant protective factors for psychological health (state-anxiety/depression).

Therefore, in line with our data, we wish to recommend that counseling interventions aiming at supporting both members of PT-infertile couples should be focused on fostering acknowledgement, reappraisal, and elaborations of the following areas: taking some time to disclose feelings about the process of prolonged experiences of treatment failures and begin, ever since the first treatment failures, an aware decision-making process on potential alternative paths (childless lifestyle; adoption); processing and reducing perceived time pressures (partially linked to age; Bühler, 2022) and perceived social concerns and pressure (partially linked to social stigma on childless couples and on adoptive-parenthood; Crawshaw and Balen, 2010; Leon, 2010); reinforcing the couple alliance by promoting affectional expression and the sharing of reciprocal concerns and ideas on infertility experience, treatment experiences, couple life, and parenting goals; promoting the recourse to more active coping strategies, so reducing the feeling of powerless and disclosing the possibility to make active and common choices.

Differently, when defining interventions targeting male and female members of QTA-infertile couples, our data suggested specific risks and resources that clinicians should take into account, i.e., the increasing number of failed treatments and the perception of high levels of need for parenthood and rejection of childfree lifestyle represented significant risk factors, while higher educational level, the recourse to problem solving (both main and moderating buffering effects) and positive attitude coping strategy, as well as the perception of couples’ dyadic consensus (both main and moderating buffering effects), represented significant protective factors for both state-anxiety and depression.

Therefore, in line with our data, we wish to recommend that counseling interventions aiming at supporting QTA-infertile couples should be focused on fostering acknowledgement, reappraisal, and elaborations of the following areas: taking some time to elaborate and process the grief and sense of losses linked to infertility and treatments failures; processing individual and couples’ feelings and perceived challenges linked to their choice to favor parenthood goal over the potential barriers of adoption (e.g., long-lasting procedure; waive the blood-tie preservation); supporting and enhancing individual and couples’ resources (i.e., couple strength as a unit; the dyadic consensus in life matters of importance and in decision-making processes; active coping profile).

Notwithstanding the potential strengths of the study, our findings should be interpreted also considering some methodological limitations. Firstly, the cross-sectional design of our study does not allow causal conclusions to be drawn. Secondly, self-report measures were used in the present study, so increasing the risk of social desirability bias. Moreover, still considering measurement tools, another study limitation concerns the adoption of the Edinburgh Depression Scale (EDS; Murray and Cox, 1990; Benvenuti et al., 1999). Indeed, the tool has been originally developed for the assessment of perinatal depression and, although there are several studies adopting it in different contexts/settings, as well as in infertility research, no validation studies of the scale within infertility population have been conducted yet. Thirdly, when analyzing/reporting moderation analysis we only relied on the statistical significance of each moderating effect along with the R-sq values, therefore, the statistically significant interaction effects we have found should be interpreted with caution, as evidence did not provide full information on how the moderator is acting in the models. Furthermore, the study was conducted in Italy, with heterosexual couples alone, so limiting the generalizability of research results. Indeed, infertility experience, childlessness, and adoptive choice are highly culturally determined and, in some cases, they are still significantly stigmatized. Future research could target same-sex couples, could address the impact of cultural dimensions, and could also be designed to compare the Italian sample with samples of infertile couples from other countries/cultural contexts. Moreover, the exploration of infertility-stress and health processes by study groups was conducted independently from the type of diagnosis and the specific type of ART treatment they have gone through. Future studies could be conducted focusing also on medical parameters in order to gain further information to be used to enhance multidisciplinary interventions. Furthermore, the present study was designed to focus and reflect upon the experiences of couples persisting in ART treatments or adopting, which represent only two out of the different paths reproductive-aged couples can go through (for example, voluntary childlessness, involuntary definitive childlessness, ART with donated gametes etc.), thus requiring further and tailored reflections to be conducted in future on a wider variety of study groups (different sub-groups of reproductive-aged couples). Finally, because of the inherently dyadic nature of infertility experience and of the choice process about parenthood, future research could be also designed to investigate infertility-related stress process by including measurement tools specifically designed to explore dyadic dimensions (e.g., dyadic coping) and by using a dyadic analytical strategy (e.g., the actor–partner interdependence model).

In conclusion, the study provides evidence to be used for the development of tailored counseling interventions targeting male and female members of infertile couples who have faced prolonged and unsuccessful repeated treatments. Promoting support interventions when infertility treatments lead to unmet goals means helping couples to undertake a complex process of decision-making to reach a shared choice fostering their wellbeing. We indeed underpinned our reflection with the initial hypothesis that infertile patients’ decision-making process may be intimately related to their mental health status. Accordingly, we suggest that exploring their unique experiences and understanding their psychological health conditions along with specific factors contributing to their mental health may also advance knowledge on the dimensions to take into account to effectively support not only their wellbeing but also their decision-making processes. Overall, for PT-infertile couples, clinicians and practitioners could use findings to guide couples towards a critical decisional point, fostering awareness of the individual, couple, and social dimensions driving the *somewhat* and *sometimes* compulsive behavior of undertaking treatments, supporting a more active attitude to face infertility experiences beyond medical treatments, and sustaining the disclosure of possible alternative paths to promote their individual and couples’ wellbeing. For QTA-infertile couples, clinicians and practitioners could use findings to effectively sustain the active elaboration of infertility grief and to follow the couple entering the new path towards parenthood, enhancing both individual and relational resources.

## Data availability statement

The raw data supporting the conclusions of this article will be made available by the authors, without undue reservation.

## Ethics statement

The studies involving human participants were reviewed and approved by Ethical Committee of Psychological Research, University of Naples Federico II (IRB:34/2019). The patients/participants provided their written informed consent to participate in this study.

## Author contributions

MZ study conception and design, interpretation of data, drafting of manuscript, and critical revision. MC acquisition of data, analysis and interpretation of data, and drafting of manuscript. FV acquisition of data, interpretation of data, and drafting of manuscript. All authors contributed to the article and approved the submitted version.

## Conflict of interest

The authors declare that the research was conducted in the absence of any commercial or financial relationships that could be construed as a potential conflict of interest.

## Publisher’s note

All claims expressed in this article are solely those of the authors and do not necessarily represent those of their affiliated organizations, or those of the publisher, the editors and the reviewers. Any product that may be evaluated in this article, or claim that may be made by its manufacturer, is not guaranteed or endorsed by the publisher.
